# The Heroic in Dental Operation, as Contrasted with Conservatism

**Published:** 1895-12

**Authors:** C. V. Kratzer

**Affiliations:** Reading, Pa.


### THE HEROIC IN DENTAL OPERATION, AS CONTRASTED WITH CONSERVATISM.[1]

BY C. V. KRATZER, D.D.S., READING, PA.

THERE may be a diversity of opinion as to what constitutes the heroic in dentistry; there may even be some scepticism as to the propriety of applying the term at all to operations in our branch of the healing art. It is not proposed here either to set up a standard or discuss the question of propriety in the application of terms.

Strictly speaking, the combination of terms in my title constitutes a paradox, for the true hero is the one who conserves the interests of his fellow-man. The subject will be viewed here, however, in a broader sense, the terms representing diametric positions, so to speak, and in this light it affords us room for argument.

The term heroism is often a misnomer for selfishness, and in no sphere, perhaps, does this apply with more aptness than in the practice of dentistry. The dentist who employs so-called heroic measures in the fearless removal, beyond the bounds of necessity, of strong and healthy tooth-structure, so that he may erect a glaring and artistic substitute of gold, is either intensely selfish or grossly deluded, for at the expense of his patient's comfort and, unwittingly, sometimes, his own vitality, he seeks to cover himself with glory, while he in no wise enhances the appearance of his subject's mouth, nor, relatively, compared with other possible means of restoration, does he truly compensate his patient or himself for the vital strain and effort.

A few weeks ago I replaced with a porcelain crown a tottering gold bicuspid contour filling of heroic proportions, which had been electro-malleted into place some eight years ago by a gold contour enthusiast. The operation was the result of seven consecutive hours of labor on the operator's part and seven hours of painful physical endurance on the part of the patient. It served the purposes of mastication for little more than seven years, and preserved the remaining tooth-structure from decay for scarcely half that time, as indicated by the extent of decay around the base, nothing but the deep-root anchorage serving to retain it. Now, the question arises, does such work pay when all the elements of time, labor, endurance, and expense are taken into account?

---

[1] Read before the Odontological Society of Pennsylvania, November 9, 1895.

Such operations are unquestionably heroic in artificial conception and extent, and yet there is a conservative idea in the leaving of weak enamel walls, a conservatism which is false, for it is exposing, rather than protecting, a weak point. A little more of the heroic in the removal of such walls, and the substitution of a gold, porcelain, or combination crown would be more durable, and avoid much labor and all the suffering and necessary endurance by the patient.

In progressive dentistry there is danger of going to extremes; there is a tendency to overdo; we are apt to get on hobbies and ride them too hard. The lamented Dr. Marshall Webb, a noble, self-sacrificing man, made this mistake; he did much for dentistry, and through dentistry for humanity; but one of the effects of his teaching has been to develop a class of contour enthusiasts; and some of these, in emulating the example of their illustrious teacher, have gone to greater extremes than even he ever dared to; and, without the skill he possessed, have made many sorry failures. We have all seen cases of gold contours in which the principle was carried to an excess which brought the filling beyond the original lines of the tooth; and this undue exposure of masticating or cutting surface, no matter even how well the work was done, would surely bring about its own destruction; such operations are certainly heroic, and, compared with the other extreme of cupped fillings, are perhaps less efficient as preservative measures. It has always seemed to me that in the building of large contour fillings, which involve the masticating surfaces of bicuspids and molars and the cutting-edges of incisors or cuspids, it is better to keep within bounds; in other words, to have the restored portion a little within the limits, as a rule, of the original outlines. This I would call conservative contour.

In prosthesis it is certainly heroic practice to excise strong and healthy incisors or cuspids so that the roots may be used for attachment of a dental bridge, but the latter can be made quite as substantial and sightly by properly fitted open-faced gold caps as anchorages, entailing the sacrifice of but little if any tooth-structure.

The heroic proportions, compared with the meagreness of its support, of some exaggerated bridge-work—or its ruins—which we have seen sometimes cause us to wonder why some men do not experiment upon the yielding sands of the ocean beach where they might drive a pile or two, and then, by building and building to this pier, learn just how far it is possible to project a cantilever over the boundless waves without crushing its support or itself. Happily, however, those who thus abuse the prosthetic idea and the principles which have been evolved from chaos for mankind's good, are largely in the minority, and belong principally to the charlatan class, which breathes a peculiar atmosphere, and is almost as distinct from legitimate dentistry as is the witch-doctor from the true physician.

Perhaps the one branch of practice which affords the best opportunity to demonstrate the difference in virtue between the heroic and the conservative, as applied to that branch, is the treatment of exposed pulps. I shall not enter into a discussion of this subject here, but the test can be made in a given case by employing the best known means of capping, and when, sooner or later, you are satisfied, by the result and inflammatory action, that there is an incompatibility in that tooth, then remove the stopping and try heroic treatment; presuming that the after-treatment is properly done, your testimony will undoubtedly be in favor of the latter, and yet there may be, and doubtless are, cases of exposed pulps which live comfortably under so-called conservative treatment for an indefinite period of time; but in all our profession I know of but one man who claims to be uniformly successful in this line.

It may not be improper to term heroic that firmness on the part of the practitioner which prompts him to insist upon pursuing that course which in his judgment the exigencies of any particular case indicate irrespective of his patient's judgment and wishes.

We are guided by our judgment in applying means of relief for whatever lesion confronts us, and firmness in our judgment inspires our patients with the necessary confidence in us.

We should employ heroic measures when necessary, and conservative when admissible; but whatever the character of our manipulations, a conservative interest in the sensibilities of our subjects should never be lost sight of; every operation should be performed as nearly without pain as it is possible with the facilities at our command. Unfortunately for us, many of our manipulations are necessarily so painful, notwithstanding the exercise of the greatest amount of care, that our patients regard us as heartless beings and themselves as the heroes, and in some cases, doubtless, their estimate is true, but to practise dentistry honestly, conscientiously, as most of us do, requires a heroism which the public is unable to appreciate. We are sometimes accused of being mercenary, of working only for gain and demanding exorbitant fees. No estimate of humanity could be more unjust. We recognize that it is not all of life to pursue a selfish end, to work and strive for self-aggrandizement, to jostle and crowd, to scuffle and tussle for worldly gain. We, as dentists, exercise our professional attainments for compensation; we expect, and sometimes receive, a fair remuneration for our services, for by that we live largely; but underneath the business surface of our professional intercourse with our patrons there is a nobler purpose, a grander aim,—a desire to give to our patients the benefit of all the skill we possess, to do for them the best we can, to work for them rather than upon them. We like to feel, when an operation is completed, that it is well done, that as a récompense for our self-sacrifice we have reached a substantial result.

There is a feeling of satisfaction in contemplating an operation well performed, an operation which gives promise to meet the intent of its building, that cannot be approached by any pleasure occasioned by the mere receipt of a money fee.

And surely this is not selfishness. Our patients' interests more than our own is the issue. We like to know that some one has been benefited by our effort, that humanity has been well served by the restoration, at our hands, of an impaired human function, and this, more than anything else, is what characterizes us as a profession,—the self-sacrificing interest in the welfare of our patients. We labor to prolong life; not our own, but that of those who come under our care.

